# Thiamine fortification strategies in low‐ and middle‐income settings: a review

**DOI:** 10.1111/nyas.14565

**Published:** 2021-01-26

**Authors:** Kyly C. Whitfield, Taryn J. Smith, Fabian Rohner, Frank T. Wieringa, Tim J. Green

**Affiliations:** ^1^ Department of Applied Human Nutrition Mount Saint Vincent University Halifax Nova Scotia Canada; ^2^ Institute for Global Nutrition University of California Davis Davis California; ^3^ GroundWork Fläsch Switzerland; ^4^ UMR‐95 QualiSud, French National Research Institute for Sustainable Development (IRD) CIRAD/IRD/University of Montpellier/SupAgro/University of Avignon/University of Réunion Avignon France; ^5^ SAHMRI Women and Kids South Australian Health and Medical Research Institute Adelaide South Australia Australia; ^6^ School of Medicine University of Adelaide Adelaide South Australia Australia

**Keywords:** thiamine, vitamin B_1_, fortification, enrichment, thiamine deficiency disorders

## Abstract

Thiamine (vitamin B_1_) is an essential micronutrient in energy metabolism and cognitive and neurological health. Thiamine deficiency disorders (TDDs) have a range of clinical presentations that result in various morbidities and can be fatal if not promptly recognized and treated, especially in infants. To intervene, thiamine intakes by breastfeeding mothers and others at risk of thiamine deficiency should be increased to ensure adequate thiamine intake. Although thiamine fortification programs have a long history in high‐income countries, there are few mandatory fortification programs to address TDDs in low‐ and middle‐income countries (LMICs), particularly in the regions of greatest concern, South and Southeast Asia. This review highlights essential aspects for consideration in the development of a mandatory fortification program in LMICs, including an overview of the data required to model fortification dosing schemes, available thiamine fortificants, and potential fortification vehicles, as well as identifies current knowledge gaps.

## Graphical abstract

There are few mandatory fortification programs to address thiamine deficiency disorders in low‐ and middle‐income countries (LMICs), particularly in the regions of greatest concern, South and Southeast Asia. This review highlights essential aspects for consideration in the development of a mandatory fortification program in LMICs, including an overview of the data required to model fortification dosing schemes, available thiamine fortificants, and potential fortification vehicles, as well as identifies current knowledge gaps.

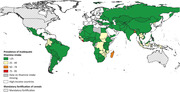

## Thiamine biology

Thiamine is an essential, water‐soluble vitamin that plays critical roles in energy metabolism and neurological and cognitive processes.^1^ Thiamine diphosphate (ThDP), the biologically active form of thiamine, acts as a cofactor in over 20 enzymatic pathways, including for pyruvate dehydrogenase and α‐ketoglutarate dehydrogenase in the Krebs cycle, and for transketolase in the pentose phosphate pathway.^2^ Impairment of these enzymes due to a lack of thiamine can impact neuronal and cardiovascular functions. For example, low acetyl‐coenzyme A production can limit acetylcholine synthesis.^3^ Finally, relatively new research suggests a vital role for thiamine in infant cognitive development,^4^ including language skills^5^, ^6^ and motor function.^7^

### Dietary sources of thiamine

The richest sources of thiamine are meat, particularly pork, and whole grains, yeast, and legumes.^1^ In many countries, major dietary sources of thiamine are fortified foods, namely refined grains, such as wheat,^8^ which are described in greater detail below. At gut concentrations lower than 1 μmol/L, thiamine is absorbed through an active, carrier‐mediated process^9^ that occurs predominantly in the jejunum and ileum,^1^ whereas passive transport occurs at higher concentrations,^9^ although intakes of thiamine above 2.5 mg are largely unabsorbed.^10^ Thiamine status can be impacted by the consumption of thiamine antagonists, such as betel nuts and tea, and thiaminase‐containing fish, ferns, and African silkworm larvae.^11^

### Thiamine requirements

Thiamine requirements are shown in Table 1. Given the role of thiamine in energy metabolism, requirements increase at times of higher energy needs. For instance, the dietary reference intakes (DRIs) in pregnancy are increased to account for both increased maternal and fetal growth and increased energy utilization.^12^ Similarly, there is higher thiamine utilization with higher carbohydrate intakes,^13^ and thus thiamine requirements increase when individuals consume high‐carbohydrate diets. The DRIs were developed for American/Canadian populations and assume that carbohydrates account for 45–65% of daily energy intake.^14^ Thus, thiamine intake should be increased if individuals or populations are known to subsist on more carbohydrate‐rich diets. At times, higher energy needs and high‐carbohydrate diets intersect, leading to thiamine deficiency. For instance, an outbreak of beriberi among young male laborers in the Gambia was attributed to physical labor during the hungry season, when workers relied heavily on imported white, polished rice to meet their increased energy requirements.^15^ Owing to a lack of evidence of adverse effects at high doses, the Institute of Medicine has not established a tolerable upper intake level (UL; the highest dose of a nutrient that is likely to pose no adverse risk) for thiamine.^12^

**Table 1:** Dietary reference intakes for thiamine

Life‐stage group	Adequate intake, mg/day	Estimated average requirement, mg/day	Recommended dietary allowance, mg/day
**Infants**			
0−6 months	0.2	–	–
7−12 months	0.3	–	–
**Children**			
1−3 years	–	0.4	0.5
4−8 years	–	0.5	0.6
**Female**			
9−13 years	–	0.7	0.9
14−18 years	–	0.9	1.0
≥19 years	–	0.9	1.1
Pregnant (14−50 years)	–	1.2	1.4
Lactating (14−50 years)	–	1.2	1.4
**Male**			
9−13 years	–	0.7	0.9
14−18 years	–	1.0	1.2
≥19 years	–	1.0	1.2

Note: Owing to a lack of evidence of adverse effects at high doses, the Institute of Medicine has not established a tolerable upper intake level (UL) for thiamine.^12^

### Thiamine deficiency

The clinical manifestations of thiamine deficiency have historically been categorized as dry beriberi (neurological presentation leading to peripheral neuropathy), wet beriberi (cardiac presentation leading to heart failure), and infantile beriberi.^16^ More recently, the term *thiamine deficiency disorders* (TDDs) has been coined to capture the broad spectrum of thiamine deficiency presentations across life‐stage groups.^11^ Thiamine deficiency presenting as Wernicke encephalopathy/Wernicke–Korsakoff syndrome is typically associated with chronic alcoholism, as reviewed elsewhere.^17^ Unfortunately, given the broad spectrum and often nonspecific nature of the clinical signs, TDDs are often misdiagnosed or mortality occurs before a TDD diagnosis is made and therapeutic thiamine is administered.^11^ Johnson *et al*. recently reviewed the prevalence of thiamine deficiency in low‐ and middle‐income countries (LMICs), noting that TDDs are most common in South and Southeast Asia.^18^

Thiamine deficiency is of highest concern among infants, as mortality can occur within a few hours of clinical presentation.^19^ Thus, pregnant and lactating women should be a priority for thiamine control and prevention programs,^11^, ^18^ as thiamine is thought to be preferentially sequestered by the fetus *in utero*,^20^, ^21^ and the thiamine intake of mothers influences milk thiamine concentrations.^22^ In addition, children with severe acute malnutrition are likely at risk of thiamine deficiency due to increased thiamine needs during critical illness;^23^ this is of particular concern during the initial phase of refeeding, with recent calls for the reformulation of therapeutic foods with increased thiamine content.^24^

## Food fortification: a brief overview

When diets are diverse and include thiamine‐rich and animal‐source foods, thiamine intakes can well exceed requirements. However, in the context of monotonous diets—in particular those lacking whole grains and meat^25^—thiamine deficiency can develop. To help prevent this, four main public health strategies exist to increase micronutrient intake: dietary diversification and modification, supplementation, biofortification, and food processing (including industrial fortification).^26^ While dietary diversification and modification is probably the most sustainable approach, if and when successfully implemented in a given context, it is very difficult to achieve, as it requires the consumer to actively change their dietary behavior. Supplementation is a faster solution, but requires constant and recurrent financial input from public health authorities if it is to be implemented at scale, so it is not sustainable in the long term. Other challenges to supplementation in LMICs include cultural acceptability, coverage and distribution, and healthcare infrastructure. Biofortification has been shown to be effective^27^ but is currently limited to a few minerals and vitamins, although varieties high in additional micronutrients (but none with high enough thiamine levels)^28^ are being developed or tested.^29^ While not yet a feasible solution for thiamine, biofortification is a sustainable approach and can, in areas that are hard to reach, complement more centralized strategies, such as industrial fortification.

Food processing, including fortification, has proven to be a very successful intervention strategy overall. In particular, large‐scale mandatory fortification of centrally processed food vehicles has been shown to reduce micronutrient deficiencies and improve health outcomes, such as reduced odds for anemia, cretinism, or neural tube defects for programs providing iron, iodine, or folic acid, respectively.^30^, ^31^ However, the impact has been more consistent for certain micronutrients over the past years (e.g., salt iodization),^32^ while for others the results have been mixed, depending on multiple factors such as the availability of suitable vehicles and bioavailable fortificants (e.g., iron fortification of wheat flour).^33^ As a whole, food fortification is viewed as a sustainable approach to address the most prevalent micronutrient deficiencies in LMICs. There is evidence that fortification has led to a substantial increase in the availability of these nutrients in the diet, with reductions in the prevalence of inadequate intakes^34^ and associated deficiency disorders.^30^

The most striking fortification success story is perhaps that of salt iodization, and the progress leading toward the reduction of iodine deficiency disorders.^35^ Throughout the 1990s, in an effort to improve iodine intakes and prevent iodine deficiency–related cognitive impairment, national governments committed to the implementation and scaling up of salt iodization policies and programs. This has led to mandatory salt iodization legislation in more than 100 countries^36^ and 88% of the world's households consuming iodized salt.^37^ Additionally, the number of countries with iodine deficiency fell from 113 in 1993 to 21 in 2019,^38^ and there have been declines in the prevalence of goiter among school‐age children, with greater reductions among populations exposed to salt iodization for a longer period of time, highlighting the long‐term success of salt iodization programs.^30^

### Past experiences of food fortification with thiamine

The first accounts of interventions to reduce thiamine deficiency consisted mainly of advocating for lower extraction rates in rice and wheat flour to maintain certain nutrient levels in the grains,^39^, ^40^, ^41^ shortly after the link between the thiamine‐rich bran and beriberi was described by the Dutch medical officer Christian Eijkman in the late 19th century.^42^ Parboiled rice was also described as being protective against beriberi and was first introduced in India in the late 1940s.^43^ Thiamine was first synthesized in 1935,^44^ allowing for its use in fortification programs.^45^ The first mandatory thiamine enrichment programs (the process of replacing nutrients lost during food processing) of white wheat flour were established in the state of South Carolina (United States) and the Dominion of Newfoundland (now part of Canada) to re‐establish thiamine, niacin, and riboflavin levels to those of whole wheat flour.^46^ The South Carolina fortification law was expanded to 26 states in the United States,^47^ and in 1976 Canada adopted a country‐wide fortification law, which mandated the addition of thiamine, along with other micronutrients, to bread and wheat flour.^45^

In Asia, the Philippines was pioneering fortification efforts with thiamine that were accompanied by a series of impact assessments in the late 1940s, demonstrating a stark reduction of thiamine‐associated mortality.^48^, ^49^, ^50^ An early large‐scale intervention in 1948 demonstrated the efficacy of thiamine‐ (and niacin‐ and iron‐) fortified rice in preventing thiamine deficiency in Bataan province, where beriberi affected 13% of the provincial population and was the second leading cause of death after tuberculosis.^48^ Two years after the introduction of the thiamine‐fortified rice, the incidence of beriberi declined to 2% in the experimental area receiving the fortified rice, compared with 9% in the control area where ordinary rice was available.^49^ In the experimental area, the beriberi mortality rate per 100,000 declined from 254 the year before the intervention to 80 during the first year of the intervention, while mortality rates in the control area remained unchanged (152 and 149 per 100,000 before and 1 year after the intervention, respectively). Deaths from infantile beriberi decreased by 52% in the experimental area during the first year of the intervention, while they increased by 5% in the control area.^49^ Of note, these data relate to presumably exclusively breastfed infants who were not consuming any rice, suggesting the effect of the fortified rice was mediated through improved maternal and milk thiamine status. Moving ahead in time to today, though fortification of certain processed foods is mandatory in the Philippines, the addition of thiamine is now voluntary.^51^

While fortification programs have proven effective in some contexts, thiamine supplements are another, sometimes complementary, strategy for the treatment as well as prevention of thiamine deficiency at the individual level or among targeted population groups, such as pregnant and lactating women, or as part of food emergencies. There was previously a high prevalence of infantile beriberi and associated mortality among Karen refugees in Thailand, so a standard food ration containing wheat flour fortified with 10 micronutrients, including thiamine, was introduced.^52^ This fortified flour had little impact on the thiamine status of lactating women or milk thiamine concentrations because of high compliance to high‐dose (100 mg/day) thiamine supplements distributed to all pregnant and lactating women in the refugee camp. Thus, fortified staple foods would likely be more effective where compliance to preventive supplementation is poor, or among isolated communities in hard‐to‐reach areas that rely on food aid distributions or government‐run safety net programs.

## When to fortify with thiamine?

**Figure 1:**
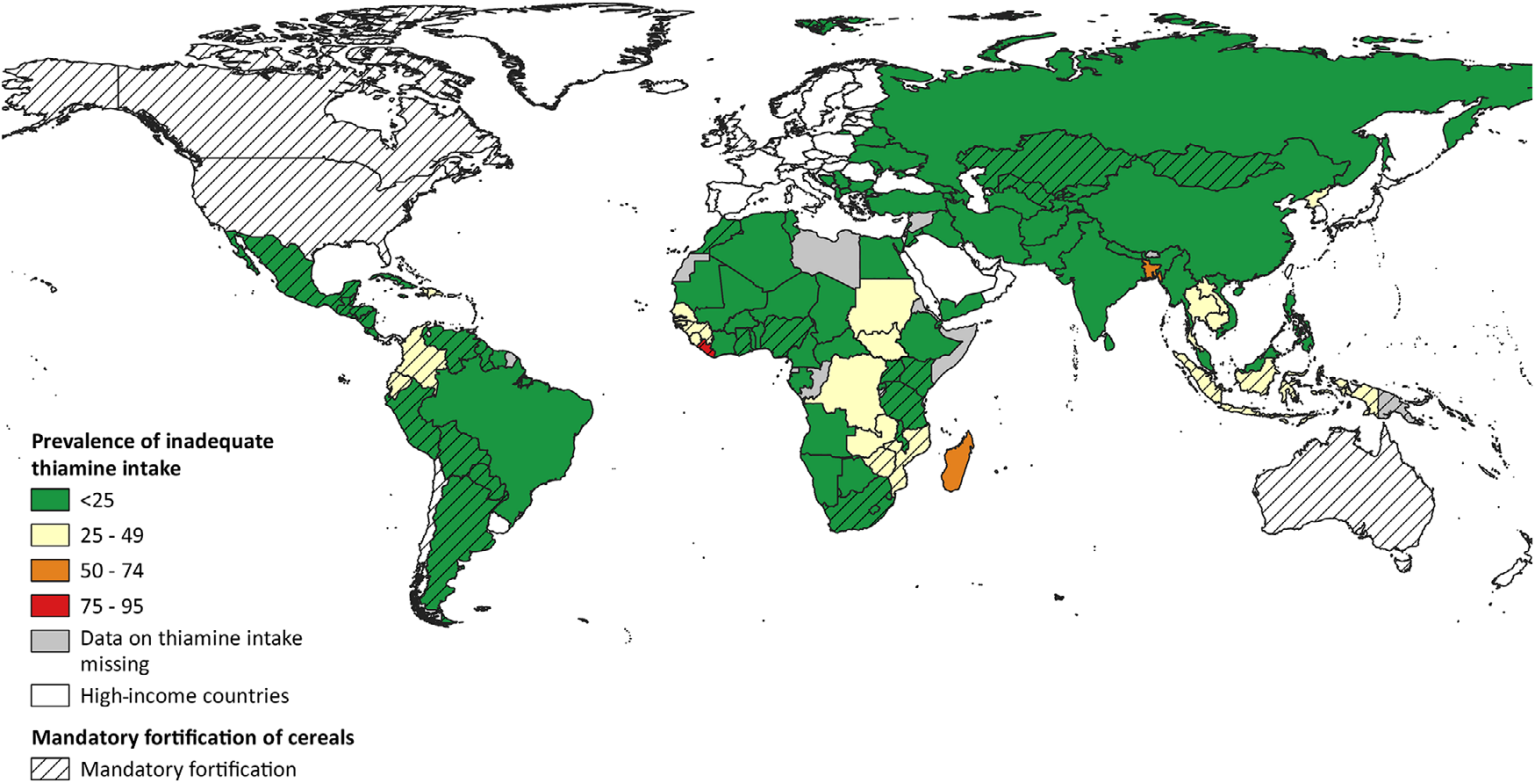
Map of prevalence of inadequate thiamine intake in 2011, not taking fortification into consideration, overlaid by whether a country has a mandatory thiamine fortification law in place for cereals. Adapted from Refs. 34 and 54.

When country stakeholders assess whether a fortification program should include a certain fortificant, such as thiamine, it is important to first establish a need, meaning that a population‐wide risk for the micronutrient deficiency should have been demonstrated. To do this, prevalence information of some sort needs to be available to decision makers, either based on dietary intake data, case reports of thiamine deficiency, or on biochemical evidence. For the case of thiamine, a recent review by Johnson *et al*. provides a comprehensive overview of available thiamine deficiency data.^18^ The review shows how diverse the nature, quality, and representativeness of the data are, rendering the task of deciding whether or not to add thiamine to an existing or new fortification program difficult for policy makers. As has previously been reviewed in detail^11^ and elsewhere in this special issue,^53^ TDDs present as a broad spectrum of nonspecific clinical signs and symptoms that often overlap with other conditions, leading to potential misdiagnosis, under‐recognition, and failure to treat appropriately. Furthermore, there are many challenges in assessing biomarkers of thiamine status in LMICs, including cost, the required cold chain, limited availability of laboratory analysis, and a lack of agreed upon cutoff thresholds indicative of deficiency.^11^ A recent publication by Beal *et al*.^34^ took the approach of estimating thiamine intakes from food balance sheets, and although this method has inherent limitations,^11^ it allows for the assessment of inadequate thiamine intake for virtually every country for which the Food and Agriculture Organization of the United Nations provides food balance sheets. In Figure 1, we show a global map indicating the inadequate thiamine intake risk by country,^34^ and we overlay a grid for those countries that currently have mandatory thiamine fortification programs for cereals.^54^ This map demonstrates how the majority of countries with a high risk of inadequate thiamine intake have no mandatory fortification programs in place. It is important to note that some of these countries may have voluntary fortification regulations, but given the mostly limited coverage of such programs, we have not considered them in this map.

As discussed above, the first step in deciding whether or not to establish a mandatory fortification program that includes thiamine is to demonstrate a public health need. Subsequently, an appropriate food vehicle needs to be identified; potential vehicles are discussed in detail below. Thiamine could be added to an existing premix for fortification programs already in place, rendering the identification of the appropriate vehicle unnecessary. If this is not the case, several previously developed tools can be employed to ensure that the selected vehicle is consumed by the majority of the population, in relatively constant quantities across the different geographies of a country or region. It is beyond the scope of this review to discuss these tools in detail, but the Fortification Rapid Assessment Tool (FRAT), developed in 2000,^55^ has been widely used.^56^, ^57^ A more recent development is that of the Fortification Assessment Coverage Tool (FACT),^57^, ^58^ which was conceptualized to assess actual coverage with fortified foods, but it can easily be adjusted for use of the potential coverage with fortifiable foods. There are other approaches, such as the Household Consumption and Expenditure Surveys data, which can be used to estimate intake of potentially fortifiable food vehicles in a population.^59^ Note that none of these tools are specific to any given micronutrient, but instead are designed to identify potentially suitable food vehicles for fortification. Once the need for thiamine fortification is established and a viable food vehicle identified, the dose modeling is required.

## Fortification dosing and requirements

The goal of fortification is to increase the thiamine intake of the target population to a level consistent with a low risk of TDDs. A manual on food fortification by Allen *et al*.^60^ and a more recent review by Allen^61^ provide detailed methods on the optimal design of food fortification programs, so that will not be repeated here. Briefly, the goal of fortification is to reduce the prevalence of inadequacy, which means shifting the target population's intake above the estimated average requirement (EAR),^60^ the amount of a nutrient that is estimated to meet the requirement for a specific criterion of adequacy for half of the healthy individuals of a particular age, sex, or life‐stage group.^14^ For thiamine, the EAR is highest for lactating women at 1.2 mg/day.^12^ Fortification exposes the entire population to thiamine; thus, the goal is to ascertain that portions of the population are not exposed to excessive thiamine. The UL, the highest level of daily nutrient intake that is likely to pose no risk of adverse health effects in almost all individuals, is often used as a cutoff for high intakes.^12^ An optimal fortification scheme is one that maximizes the number of people in the target population meeting the EAR while minimizing the number above the UL.^60^ Even though there is no UL for thiamine, this does not mean there is no potential for adverse effects resulting from high intake, and thus the lowest effective dose of thiamine should be used.

### Determining current intakes of thiamine and potential fortification vehicles

As described above, there are several pieces of information required to design any thiamine fortification scheme. An estimate of current thiamine intakes and its distribution in the target and nontarget populations is required. Likewise, an estimate of the amount of the food vehicle consumed is required along with its intake distribution. Although food balance sheets can be used, these estimates are best obtained through a (repeated) 24‐h recall in a survey in a random sample of all population subgroups. When this is not possible, a survey of the target population and those at highest risk of excess, typically pregnant/lactating women and men, respectively, should be conducted. A detailed explanation of how to conduct surveys and 24‐h recalls is provided in a manual by Gibson and Ferguson,^62^ and the thiamine contents of African and Asian foods are available in Food Composition Databases.^63^ A single recall tends to result in a wide distribution with more people at the extremes of intake, both high and low. A repeat recall in a subset of participants allows for statistical adjustment of intrasubject variation and represents a more “usual” intake distribution. This results in a smaller proportion of the population below the EAR or above the UL.^64^ The same adjustments should be made for the fortification vehicle. There are a number of statistical approaches used to adjust intakes for intrasubject variation and software is freely available, for example, the Software for Intake Distribution Estimation (PC‐SIDE) developed at Iowa State University.^65^, ^66^

### Fortification modeling

**Figure 2:**
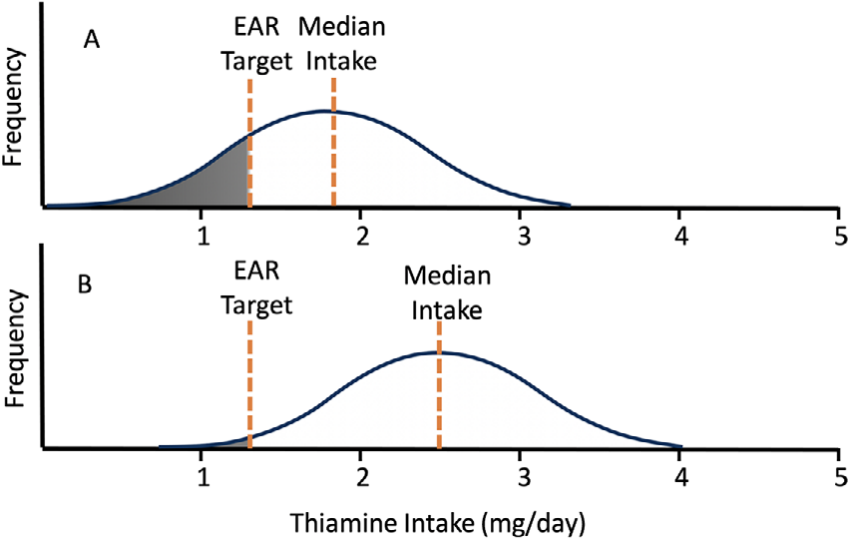
A hypothetical distribution of thiamine intakes in women of reproductive age before and after fortification. The target intake for thiamine was set at 1.2 mg, the estimated average requirement (EAR) for pregnant/lactating women. In panel A (prefortification), 30% of women are below the EAR and the median thiamine intake is 1.8 mg/day. In panel B (postfortification), only 2.5% of women are below the EAR, and the median intake is 2.5 mg/day. Adapted from Ref. 60.

With knowledge of the intake distributions of both thiamine and the fortifiable food vehicle, it is possible to estimate the amount of thiamine to be added.^61^ No fortification scenario will allow all of the target population to achieve the EAR for thiamine if they do not eat the chosen food vehicle, or eat so little of that food that the level of thiamine required would cause high consumers of that food to be exposed to excessive intakes of thiamine. Generally, a fortification scheme should allow all except 2–3% of the population to meet the EAR, and no more than 5% of the population to exceed the UL, if one exists. The Intake Monitoring, Assessment, and Planning Program (IMAPP) software for Intake Distribution Estimation developed by Iowa State University allows for adjustment for intrasubject variation of both the nutrient and food vehicle.^67^ Various scenarios can be modeled for the addition of thiamine to various food vehicles to determine the percentage of the target population meeting the EAR. IMAPP uses the current nutrient intake distribution for a population subgroup, the distribution of intake of the vehicle, and uses various levels of added thiamine to develop a new distribution that models the proportion of the population below the EAR. IMAPP can also be used to determine the percentage of the population above the UL.^61^ Figure 2 shows a hypothetical scenario indicating how fortification could reduce the prevalence of thiamine inadequacy.

## Thiamine fortificants

Compared with other micronutrients, thiamine has few constraints in the categories of technological issues, sensory challenges, safety (given the lack of a UL), and cost.^60^ The two thiamine salts commonly used in fortification are thiamine hydrochloride and thiamine mononitrate. Both are heat labile,^60^ and also sensitive to humidity, oxygen exposure, the presence of sulfites, and radiation.^68^ Thiamine is most stable in acidic conditions below approximately pH 6.^68^ Thiamine hydrochloride is used in liquids because of its high solubility, whereas the low hygroscopicity of thiamine mononitrate makes it better suited for use in dry products.^68^ Thiamine is often described as nearly odorless;^69^ however, thiamine degradation products can produce aromas. In a forced choice sensory panel, untrained panelists identified thiamine hydrochloride more often than thiamine mononitrate as having a strong smell; however, researchers used samples unlikely to be used in fortified products: 5 mg/mL samples heated at 80 °C for 48 h before sensory evaluation.^68^

Benfotiamine is a synthetic, lipid‐soluble thiamine analog^70^ that could be used to fortify oils or oily foods. The bioavailability of orally administered benfotiamine is higher than both thiamine hydrochloride^71^ and other thiamine formulations commonly used in clinical settings (thiamine disulfide and fursultiamin).^72^ However, benfotiamine is not currently used in fortification.

## Potential thiamine fortification vehicles

In selecting a vehicle for thiamine fortification, there are several important considerations. Ideally, the food should be consumed by the target population daily. In most countries, this means selecting a food staple, such as rice or wheat flour, or condiments, such as sugar or salt.^61^ This will be context‐specific; in many high‐income countries, wheat flour is an ideal choice because many people eat bread daily,^73^ but in many LMICs, rice is more commonly consumed.^74^ Ideally, variation in intake of the vehicle should be similar across sex and life‐stage groups. In reality, this is rarely achieved as consumption of the food vehicle often varies with energy intake. Consider thiamine fortification where the aim is to increase intake among women of reproductive age; if rice were selected as a fortification vehicle, men or adolescent boys with higher energy requirements will consume more rice and, therefore, more thiamine than women, the target population who have lower energy requirements and consume less rice.^61^ Similarly, food consumption trends should be monitored over time, and fortificant doses modified to align with current intakes, as is currently encouraged with salt reduction programs in countries with mandatory iodization.^75^ Another consideration is whether the food vehicle is centrally produced.^60^ Wheat is often milled at one or two large mills in a country, whereas rice may be milled at the community level. The other issue is the technical feasibility of fortifying the vehicle; foods like flour or salt may be technically easier to fortify than whole grains.^76^ Of note, thiamine fortification nearly always occurs as part of a multiple micronutrient fortification program, so investigating cofortification of a vehicle already in use in‐country is warranted.

### Wheat flour

Thiamine is mainly found in the bran and germ of the wheat kernel, with up to 50% of it lost during milling.^77^ Hence, fortification of wheat flour with thiamine can be seen as an attempt to restore thiamine levels (enrichment), although current fortification levels are often much higher than the original concentrations present.^78^ Today, mandatory wheat flour fortification is in place in over 85 countries, but several of these countries do not require thiamine to be included in the premix. For example, Nepal only stipulates the addition of vitamin A, iron, and folic acid to wheat flour. Of the Southeast Asian countries where beriberi has historically been identified, only Indonesia has mandatory fortification of wheat flour with thiamine. However, this is likely because wheat flour fortification is less attractive in some countries as wheat flour is mainly used by the wealthy in the population^79^ or is not a main staple food. For instance, in Guatemala, there was a 15‐fold difference in fortified wheat flour consumption between the poorest and richest groups (7 and 110 g/day, respectively).^80^ Similarly, in the Solomon Islands, consumption of fortified wheat flour by women of reproductive age was estimated at only 22 g/day, contributing to only 15% of the EAR for thiamine.^81^ By contrast, rice fortified with thiamine at typical levels would fulfill > 100% of the EAR for thiamine for women of reproductive age.^81^

Thiamine and other micronutrients are normally added directly to the flour after milling, typically with overages to compensate for the losses during food processing. Previously, the heat exposure during bread baking was thought to cause thiamine degradation, with losses estimated between 15% and 20%.^60^ However, newer research from Australia has shown that although baking results in some thiamine losses, these are more than made up for by the addition of yeast in the bread‐making process.^82^ Tiong *et al*. demonstrated that, through the addition of naturally thiamine‐rich yeast, both white and wholemeal bread dough reached peak thiamine concentrations, which fell only slightly with baking, yielding a bread with higher thiamine than the flour itself.^82^

There is a wide range in the mandatory fortification levels of thiamine, ranging from 1.5 to 11 mg/kg of wheat flour.^60^ The higher end of this range should not pose any health risk, as there is no known toxicity for thiamine, but as noted above, ideally the lowest effective dose of thiamine should be used. Also, commercial products made from wheat flour tend to be lower, rather than higher, in thiamine content. For example, in Australia, commercial breads made with fortified wheat flour were at the lower end of the mandatory range.^82^

### Rice

Rice is the second most commonly consumed grain globally. In some countries, rice accounts for as much as 70% of energy intake.^83^ Given rice's near‐universal consumption, especially in countries where TDDs exist, it is an ideal thiamine fortification vehicle from a consumption coverage standpoint. Curiously, brown rice is a good source of thiamine and several other nutrients; however, in the process of polishing rice, most of the micronutrients, including thiamine, are stripped away with the bran and germ. Rice can also be fortified with multiple micronutrients simultaneously. Unlike other fortification vehicles, such as flour and salt, rice is consumed as an intact kernel, making fortification technically challenging.

The three leading technologies used to fortify rice are dusting, coating, and extrusion.^76^ In high‐income countries, dusting is the most common process used to fortify rice. With dusting, a dried powder is added to the milled rice, which adheres to the kernels through electrostatic forces.^76^ Unfortunately, in many LMICs, rice is washed, soaked, and cooked in excess water that is discarded before consumption, making dusting unsuitable. Coating involves spraying the rice with a liquid that contains the micronutrients and other ingredients, such as waxes or gums, that allow the nutrients to better adhere to the rice kernels. These coatings need to be resistant to washing and soaking, but need to dissolve during cooking to release the micronutrients into the cooked rice. Extrusion involves grinding rice into a flour, combining it with the micronutrient mix and water, and reshaping it into a rice kernel followed by drying. These fortified kernels are blended with nonfortified rice in a ratio required to meet the desired fortification level, usually between 0.5% and 2%.^76^ A drawback of this method is that the extruded kernels may look slightly different from unfortified rice kernels, so consumers may remove them thinking they are spoiled.

The amount of thiamine retained in cooked rice varies by fortification method, as well as cooking method and duration. Thiamine is water‐soluble and is leached into the cooking water where it may undergo thermal degradation.^84^ Cooking techniques that minimize water content losses, such as microwaving, stir‐frying, and pressure‐cooking, result in higher thiamine retention. However, boiling is the primary method of cooking rice in low‐income settings. Thiamine losses after 12 min of boiling were reported to be 50% when rice was fortified via soaking in a vitamin solution at 90 °C for 15 min followed by oven‐drying, compared with only 10% with rice fortified by extrusion.^85^, ^86^ Extra thiamine can be added to rice (fortification overages) on account of these losses.

Rice fortification requires large centralized mills and a significant investment in equipment. A disadvantage of rice fortification is that it is not possible where rice is grown and milled at the household or community level, as this introduces challenges of premix distribution and quality control.^87^ For example, factors instrumental to the success of a rice fortification program in Costa Rica included government leadership and political will for legislative monitoring and enforcement, private sector support, controlled consumer prices and consumer acceptability, strong emphasis on the importance of monitoring and compliance, a centralized rice industry, and engaging and leveraging existing distribution channels.^88^ Rice milling in other countries, particularly in Southeast Asia (e.g., Cambodia, Laos, and Myanmar), is often decentralized and more fragmented, making implementation complex. For example, in Laos, it is estimated that there are >35,000 rice mills, of which approximately 25,900 are custom mills with small capacity, and there are very few (<10) mills with large capacity.^89^ In Myanmar, only around 15 out of >2000 rice millers are engaged in the rice fortification program, which would reach only 1% of the total population of Myanmar.^11^

### Salt

Salt iodization occurs in over 100 countries,^36^ and with a reach to over 70% of the world's population and concurrent improvement in iodine status since the 1990s, it is considered a successful public health initiative.^35^ Salt is a ubiquitous condiment, consumed by people of all economic classes, in all countries, throughout the year, in consistent quantities.^90^, ^91^ Importantly, salt intakes tend to be less variable between sex and life‐stage groups because it is not as closely linked to energy intakes. In addition, iodization technology for spray fortification with liquid potassium iodine premix solution is widely available and inexpensive.^90^ Given the success of iodization, cofortification of salt with other micronutrients is considered an ideal means of piggybacking on existing infrastructure and public health messaging. Salt cofortified with iodine and thiamine was recently modeled with lactating Cambodian women as the target population (see elsewhere in this special issue).^92^ However, to date, no iodization programs have cofortified iodine and thiamine in salt produced using current spray fortification technologies, although this intervention seems feasible as undesirable reactions between iodine and thiamine are unlikely.

Instead, much research has focused on cofortification of iodized salt with iron as a means of combatting two major deficiency disorders, iodine deficiency disorders and iron deficiency anemia.^91^ However, since iron compounds have a strong taste and color, and ferrous iron compounds react adversely with potassium iodine, iron must be microencapsulated, rather than spray‐fortified, in salt fortified with multiple micronutrients.^91^ This is a more expensive fortification process, largely due to the need for new fortification mixing equipment to combine the iron premix with spray‐iodized salt.^91^ Salt fortified with multiple microencapsulated micronutrients, including thiamine, was previously evaluated as part of an Indian school feeding program; however, biochemical indicators of thiamine were not measured.^93^ Quadruple‐fortified salt premix containing microencapsulated iodine, iron, vitamin B_12_, and folate has been piloted in India as well.^94^ Although iodine‐thiamine cofortification can be done using simple spraying techniques, thiamine can easily be incorporated in the context of more advanced microencapsulation technology.^93^

In either case, quality control programs to monitor and enforce fortification are a vital component of a successful salt fortification program.^95^ A point‐of‐care thiamine assessment tool or kit is not available at the moment, but would be extremely helpful in facilitating rapid quality inspection, and development has been identified as a research priority.^11^ Alternatively, if the spray premix contains both iodine and thiamine, use of current field tests for iodine^96^ could continue if enforcers could reasonably assume that any premix containing iodine would also contain thiamine.

The World Health Organization is currently promoting a global salt reduction strategy, with recommendations to reduce population‐level salt intake to 5 g salt/day.^75^ Salt reduction strategies and iodine‐deficiency prevention programs, namely salt iodization, are entirely compatible. As salt consumption patterns decline, it is essential that salt fortification efforts are aligned, which can be achieved through robust monitoring and evaluation, including review and adjustment of fortification standards as needed, such as increasing micronutrient levels in all food grade salt as salt intakes decline, to ensure the intended impacts are sustained and safely met.^75^

### Other fortification vehicles

It is possible to fortify other food staples, such as sugar and vegetable oil with thiamine, but at present, these staples have only been used for vitamins A and D at scale.^97^, ^98^ Also, condiments other than salt, including fish sauce, soy sauce, bouillon cubes, and curry powders, could be used as fortification vehicles for thiamine.^99^ In particular, fish and soy sauces are commonly consumed condiments across various age, sociodemographic, and geographic groups in Asia due to their wide accessibility and affordability. In Cambodia,^100^ Thailand,^101^ and Vietnam,^102^, ^103^ fish sauces are fortified with iron, and in Thailand fish sauce is voluntarily doubly fortified with iron and iodine.^101^ However, soy sauce was recently found not to be consumed frequently in rural Cambodia,^92^ despite current iron fortification efforts in the country. In efficacy studies, thiamine‐fortified fish sauce was shown to increase blood biomarkers in Cambodian women and children, including infants of lactating mothers.^104^, ^105^ However, condiment use varies by country, and there are often many producers, making enforcement difficult.^106^ For example, in Cambodia there are over 40 fish sauce producers,^100^ and home production of fish sauce occurs, particularly in rural areas. Thus salt, a significant ingredient in fish sauce, would be a better fortification vehicle than fish sauce itself. In Asian countries, prepackaged noodles are often fortified with thiamine voluntarily, as are many ready‐to‐eat breakfast cereals; however, these are often premium brands, which usually cost more, and hence do not reach the most vulnerable populations.

In several countries, including South Africa, Australia, and New Zealand, the fortification of beer with thiamine has been promoted to prevent Wernicke–Korsakoff syndrome in alcoholics.^11^, ^107^ Indeed, fortification of beer with thiamine is rather straightforward, with fortification levels of 2.5 mg/L assuring attainment of the EAR.^108^ However, the Australia New Zealand Food Standards Code does not permit the fortification of alcoholic beverages. Thiamine fortification of wine appears to be ineffective, as within a year most, if not all, of the thiamine added (80−100%) was lost, most likely due to sulfur dioxide present, especially in white wines.^109^ Additionally, sulfites cleave the pyrimidine ring from the thiazole ring.^110^ Other drinks that have been fortified with thiamine include tea and coffee, but tannic acid oxidizes the thiazole ring, thereby preventing thiamine absorption, making fortification of these beverages with thiamine ineffective.

Micronutrient powders (MNPs), single‐use sachets containing a dry mix of micronutrients, can be used for home, or point‐of‐use, fortification.^111^ MNPs are used most often to improve the micronutrient intakes of young children aged 6–23 months by mixing the sachet with complementary foods during this period of vulnerability for micronutrient deficiencies.^111^ Thiamine is routinely included in MNP formulations at the recommended nutrient intake for children aged 6–23 months, which is 0.5 milligrams.^112^ Although MNPs have been used among population subgroups beyond the complementary feeding period (e.g., pregnant and lactating women),^112^ given the breadth of insufficient thiamine intakes in regions where thiamine deficiency occurs,^18^ mandatory staple‐food fortification is likely a more sustainable solution.

### The importance of local context in fortification vehicle selection: cultural dietary practices

When establishing food fortification programs, special consideration may need to be given to local or regional traditional dietary practices. For example, highly restrictive diets during the perinatal period are common in many Southeast Asian countries.^113^, ^114^ It has previously been reported that 80–98% of mothers adhered to postpartum food restrictions in Laos,^115^, ^116^, ^117^ 96% in Myanmar,^118^ 79% in Thailand,^119^ 60% in Cambodia,^120^ and 28% in Indonesia.^121^ Restrictive diets during pregnancy are less common, but the practice of “eating down” to avoid the perceived difficult delivery of a large baby has been reported.^117^, ^122^, ^123^

Commonly reported reasons for perinatal food avoidances, determined through qualitative research, include helping the mother's body to heal after childbirth, to avoid illness in the mother and her child, and to promote adequate quality and quantity of breastmilk,^113^, ^114^, ^117^, ^123^ although mothers have also stated that food taboos are passed onto them from close family members for reasons unknown.^117^, ^123^ Postpartum diets are often the most restrictive in the first 2 weeks to 1 month after childbirth, with some women in Laos limiting their diet to just rice and salt, and a tea made from herbs and roots.^115^, ^117^, ^124^ Vietnamese mothers reported that salty foods are regularly consumed as it helps to ensure sufficient breastmilk quantity.^125^ Fish sauce during pregnancy was “not good” according to Laotian women^117^ and was avoided during lactation by mothers in Laos and Cambodia due to negative influences on the health of the infant and beliefs that it is harmful to breastfeeding women and avoiding it would ensure adequate breastmilk.^115^, ^117^, ^126^ Sauces were often avoided in the first few months postpartum by women in Vientiane Capital, Laos.^115^ After the initial highly restrictive phase, other foods are consumed, predominantly rice with poultry, certain meats or fish, and limited vegetables.^115^, ^117^ Postpartum food restrictions can continue from 2 days to 2–3 years after delivery. These highly restrictive diets likely contribute to multiple micronutrient deficiencies in mothers that may have important consequences for their breastfed infants through reduced micronutrient content of breastmilk. For example, in a cross‐sectional study of 300 mothers of infants <6 months of age in Laos, 97% of mothers had inadequate intakes of thiamine (among other micronutrients), although this did not differ between women adhering to a restrictive diet compared with those who were not.^115^ However a limitation of that study was the use of a single 24‐h dietary recall and the lack of breastmilk and blood biochemical indicators of micronutrient status, which has yet to be explored.

Given the prevalence of restrictive diets and food taboos during the perinatal period in many LMICs,^127^ it is critical to understand traditionally consumed and restricted foods and/or condiments when selecting fortification vehicles. Although these diets are deeply rooted cultural traditions and are prevalent across all regions and ethnic groups within countries, specific practices and food avoidances are not always consistent between or even within different villages or ethnic groups, posing a significant challenge to the identification of a food vehicle. For instance, fish or other sauces may be consumed in one region of a country or by certain ethnic groups, but not in another; therefore, fortifying sauces would not reach pregnant and/or lactating women if these are the intended target population. Alternatively, supplementation may be more appropriate during the perinatal period, although it is yet to be determined if supplements are culturally acceptable during this highly restricted period, and this requires further exploration.

## Quality control/quality assurance considerations for fortification

For food fortification to have an impact, the fortified product should be accessible to the target population and be consumed frequently and in regular quantities. However, it is also important that the product reach the target population with the expected quality, including the targeted micronutrient content. To ensure this, a comprehensive yet feasible quality control and quality assurance system needs to be established from the point of food production to the level of the end consumer. Mandatory food fortification with thiamine, regardless of whether it is single or multiple micronutrient fortification, should undergo the same quality control procedures as other fortification programs. The 2006 publication “Guidelines on food fortification with micronutrients” provides a succinct summary of the components required to monitor and evaluate a food fortification program,^60^ and although there have been more comprehensive descriptions of the system for flour fortification,^128^ the principles remain the same, including for rice fortification.^129^

In brief, monitoring of fortification programs can be described as follows: (1) monitoring the supply and quality of fortified foods, and (2) assessing the availability, utilization, coverage, and reach of fortified foods.

### Monitoring the supply and quality of fortified foods

This element of the quality control and assurance process ensures that the food is produced according to pre‐established quality criteria, and is often referred to as regulatory monitoring. The regulatory monitoring component is mainly composed of an internal and an external check. The internal monitoring relates to the quality assurance and quality control conducted by the suppliers of premix and producers of fortified foods to ensure that the product complies with certain criteria when the food product leaves the factory; an important aspect of the internal monitoring is complete and clear documentation of critical control points, since this is the first entry point for the external monitoring. External monitoring is typically implemented by the government or a government‐mandated entity, and consists of factory inspections and audits. Here, thorough documentation of quality assurance and control on the side of food producers is crucial as an entry point. The independent testing of the quality of the food product is also part of external quality control. Detailed toolkits for internal and external monitoring have been developed for flour and rice by A2Z^130^ and the Food Fortification Initiative (FFI),^131^, ^132^ and FFI also developed a management information system for fortified foods to help industries systematically collect information.^133^ Furthermore, a critical assessment of challenges and potential solutions has been conducted by Luthringer *et al*.^134^

Import monitoring also falls under this category of ensuring that the fortified foods reach the end consumer with adequate quality, and although there are general guidelines for import inspection available,^135^ more specific guidance for fortified foods are mostly adapted for a country or a region.^136^, ^137^

Also typically considered part of the regulatory monitoring, although most often conducted by different actors, is commercial monitoring, whereby wholesale and retail compliance with food regulations are ensured.^60^ Some country examples have been compiled by the FFI.^138^

### Assessing the availability, utilization, coverage, and reach of fortified foods

This part of the monitoring system is concerned with the local availability of fortified foods, the effectiveness of social marketing and communication efforts, and utilization and consumption of fortified foods at household or individual level. This kind of assessment is typically composed of cross‐sectional surveys and can be complemented by qualitative research to better understand barriers and enablers affecting adoption within the target population. The FACT is a tool that was developed to assess this kind of information.^58^

## Research gaps and outlook

It was nearly a century ago that thiamine was characterized and quickly added as a fortificant to staple foods as a public health intervention in certain countries. It is, therefore, surprising that today still so many aspects of TDDs remain unknown. Even though dietary intakes of thiamine appear to be sufficient in many countries in Africa and Asia (Fig. 1), subgroups of populations in these countries may be at risk for thiamine deficiency. In order to appreciate the true global health impact of TDDs, better data on thiamine intake and the status of populations should become available. Unfortunately, too much focus is often placed on a few select micronutrients, and even if national micronutrient surveys are being conducted, thiamine is rarely included. For those countries where thiamine status is known to be a public health issue, efforts should be made to identify suitable food vehicles for fortification. As discussed in the present paper, this might require some innovative solutions. Reconstituting thiamine levels in foods to the original level before food processing might seem the most logical approach; for example, to restore thiamine levels in rice to those as before milling. However, this approach is often impractical due to the nature of the agriculture and food systems in countries where thiamine deficiency is prevalent, with households or communities processing their own rice. Therefore, finding alternate food vehicles for thiamine fortification that are consumed by the most vulnerable groups is essential. Dual fortification of salt with iodine and thiamine is an option that could be implemented in numerous settings in the near future, and more research should be directed toward this intervention. Research questions to be addressed include best practices for adding thiamine (together with iodine) to the salt, guaranteeing stability of thiamine over time, and, as for all fortification programs, determining how best to monitor the implementation and impact. Beyond the scope of this review on thiamine fortification, more research into the role of thiamine deficiency in the cognitive development of children is urgently needed, as is more research to determine the best biomarker and relevant cutoffs for thiamine status. Fortunately, thiamine is back on the research agenda, after having been absent for almost 50 years. We can no longer ignore that thiamine deficiency is an important determinant of public health in many LMICs, and large‐scale, mandatory food fortification with thiamine seems the most cost‐effective strategy to address this pressing issue.

## Author contributions

All authors contributed to writing this review, and all authors read and approved the final manuscript.

## Competing interests

The authors declare no competing interests.
